# Motivational Interviewing and Glycemic Control in Adolescents With Poorly Controlled Type 1 Diabetes: A Randomized Controlled Pilot Trial

**DOI:** 10.3389/fendo.2021.639507

**Published:** 2021-03-12

**Authors:** Anna-Kaisa Tuomaala, Matti Hero, Martti T. Tuomisto, Maria Lähteenmäki, Päivi J. Miettinen, Tiina Laine, Karoliina Wehkalampi, Sanne Kiiveri, Pekka Ahonen, Marja Ojaniemi, Kari Kaunisto, Päivi Tossavainen, Risto Lapatto, Taisto Sarkola, Mari-Anne Pulkkinen

**Affiliations:** ^1^ Children’s Hospital, University of Helsinki and Helsinki University Hospital, Helsinki, Finland; ^2^ Faculty of Social Sciences (Psychology), Tampere University, Tampere, Finland; ^3^ Department of Pediatrics and Research Unit for Pediatrics, Pediatric Neurology, Pediatric Surgery, Child Psychiatry, Dermatology, Clinical Genetics, Obstetrics and Gynecology, Otorhinolaryngology and Ophthalmology, Oulu University Hospital and University of Oulu, Oulu, Finland; ^4^ Minerva Foundation Institute for Medical Research, Helsinki, Finland

**Keywords:** adolescent, diabetes, glycemic control, motivational interviewing (MI), HbA1c

## Abstract

A multicenter randomized controlled pilot trial investigated whether motivational interviewing (MI) by diabetes physicians improves glycemic control and variability in the context of follow-up for adolescent patients with poorly controlled type 1 diabetes. Patients (*n* = 47) aged 12 to 15.9 years who showed poor glycemic control (HbA1c >75 mmol/mol/9.0%) were randomized to standard education (SE) only or MI+SE, with study physicians randomized to employ MI+SE (*N* = 24 patients) or SE only (*N* = 23). For one year of follow-up, the main outcome measurements were obtained at three-month visits (HbA1c) or six-monthly: time in range (TIR) and glycemic variability (CV). Mean adjusted 12-month change in HbA1c was similar between the MI+SE and SE-only group (-3.6 vs. -1.0 mmol/mol), and no inter-group differences were visible in the mean adjusted 12-month change in TIR (-0.8 vs. 2.6%; *P* = 0.53) or CV (-0.5 vs. -6.2; *P* = 0.26). However, the order of entering the study correlated significantly with the 12-month change in HbA1c in the MI+SE group (*r* = -0.5; *P* = 0.006) and not in the SE-only group (*r* = 0.2; *P* = 0.4). No link was evident between MI and changes in quality of life. The authors conclude that MI’s short-term use by diabetes physicians managing adolescents with poorly controlled type 1 diabetes was not superior to SE alone; however, improved skills in applying the MI method at the outpatient clinic may produce greater benefits in glycemic control.

## Introduction

During adolescence or young adulthood, poor glycemic control markedly increases the incidence of later micro- or macro-vascular complications in type 1 diabetes patients. Treatment adherence often declines in youth (1). Simultaneously, pubertal hormonal changes are known to affect metabolism and insulin needs, making diabetes management even more complicated (1–4). At present, technical advancements in insulin delivery and glucose monitoring fail to address the challenges in self-care during adolescence. Therefore, evidence-based and reliably adoptable methods to improve treatment adherence are necessary in the management of adolescents with poorly controlled type 1 diabetes.

Health-care professionals have used motivational interviewing (MI) for various disorders and behavioral problems, among them alcohol and drug problems, gambling, and cardiovascular diseases (5). MI is a counseling approach designed to facilitate activity-elicited or automatic motivation in people to change their behavior. Previous studies show that MI improves commitment to care when supplementing other treatments (6). However, only a few studies have evaluated the benefit of MI alongside standard care in the follow-up of adolescents with type 1 diabetes. Furthermore, findings have been inconsistent, from substantial benefit (7, 8) to neutral effect (9–12). In these studies, MI was applied in variable populations and settings (7–12). Accordingly, while reporting a limited impact of MI overall, a meta-analysis of MI in the management of glycemic control in diabetes, as judged by HbA1c levels, recommended further research examining delivery and focus of MI (12). So far, it remains unclear whether MI could yield benefits in the outpatient clinic setting.

We hypothesized that MI added to standard education care would improve glycemic control in adolescents with poorly controlled type 1 diabetes. We conducted a randomized controlled pilot trial to evaluate whether the use of MI indeed is associated with decreased HbA1c levels and improves glucose variability in poorly controlled cases of adolescents with type 1 diabetes. Our secondary aim was to study MI’s effect on health-related quality of life.

## Materials and Methods

**Figure 1:**
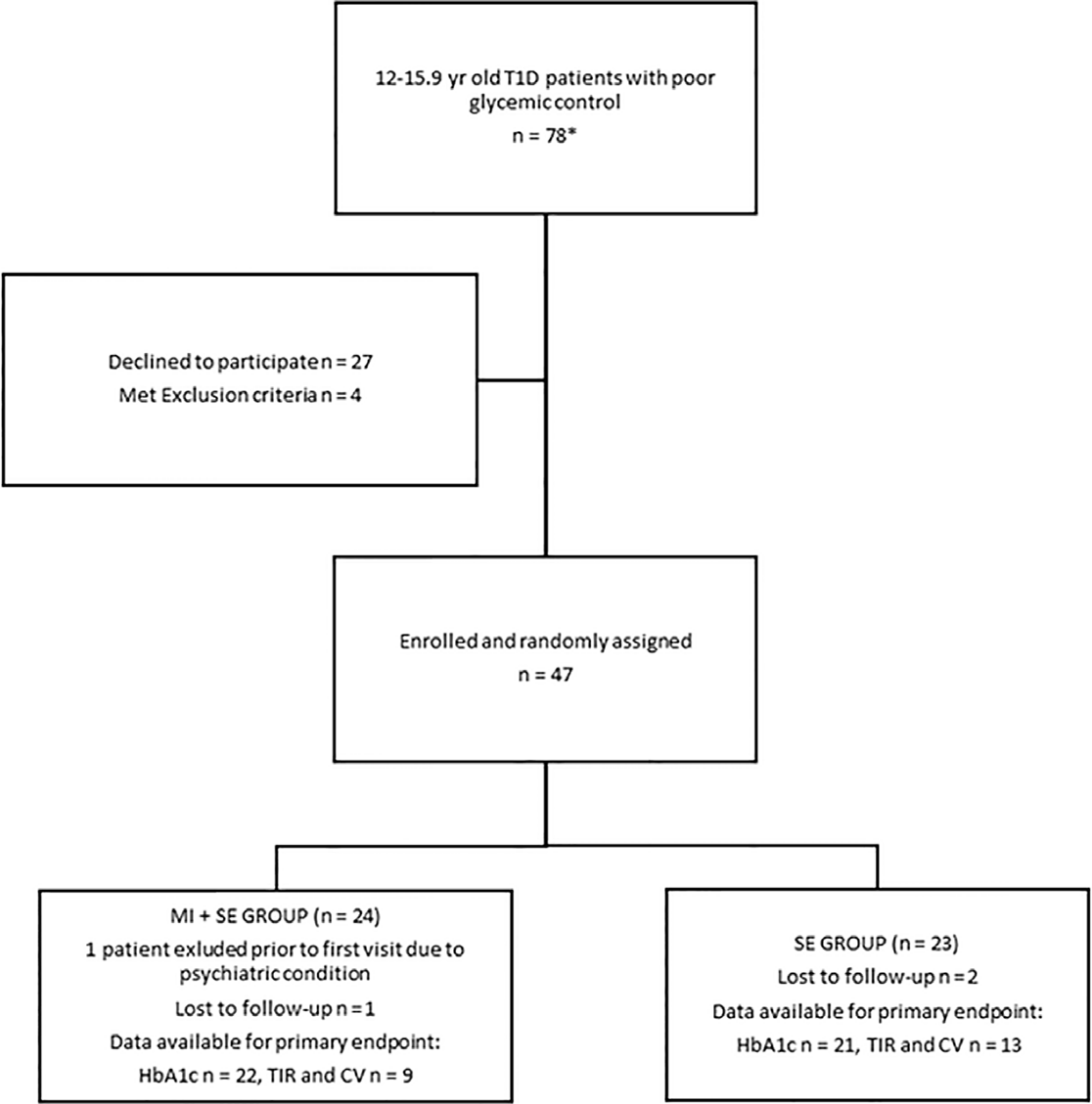
Study Flowchart.

The participants were recruited from three large tertiary care outpatient pediatric diabetes clinics in Finland (two at Helsinki University Hospital and one at Oulu University Hospital) between September 2015 and September 2017. These outpatient clinics follow approximately 1,300 patients with type 1 diabetes who are below 17 years of age, accounting for 25% of all pediatric type 1 diabetes patients in Finland. Pediatric type 1 diabetes patients attend publicly funded health care in Finland with a nominal outpatient clinic fee. The study protocol was integrated into clinicians’ daily practice as part of regular clinic visits. The **inclusion criteria** were 1) at least two years duration of type 1 diabetes, 2) HbA1c > 75 mmol/mol/9% on the two immediately previous visits, 3) an age of 12.0–15.9 years, and 4) being at Tanner pubertal stage 2 or above at enrollment. The **exclusion criteria** were celiac disease with poor control, diagnosis of a severe psychiatric disorder, and chronic disease requiring systemic glucocorticoid treatment. Patients who met both the HbA1c and the age criterion were screened from the hospital database and records, and their treating physicians consented to participation in the study. In all, 78 randomly recruited subjects of Caucasian origin fulfilled the study’s inclusion criteria, of whom 47 (21 female) were enrolled and randomized (see **Figure 1**) 1:1 to the intervention group (MI+SE) or the control group (SE). Randomization was performed in permuted blocks of six patients with balanced numbers of intervention and control subjects in each block. The Helsinki University Hospital Committee on Medical Research Ethics approved the study protocol, and the Good Clinical Practice principles and the terms of the Declaration of Helsinki were followed. Informed consent was obtained in writing from all participants and their guardians. The study is registered with ClinicalTrials.gov (NCT02637154).

### The Intervention

**Figure 2:**
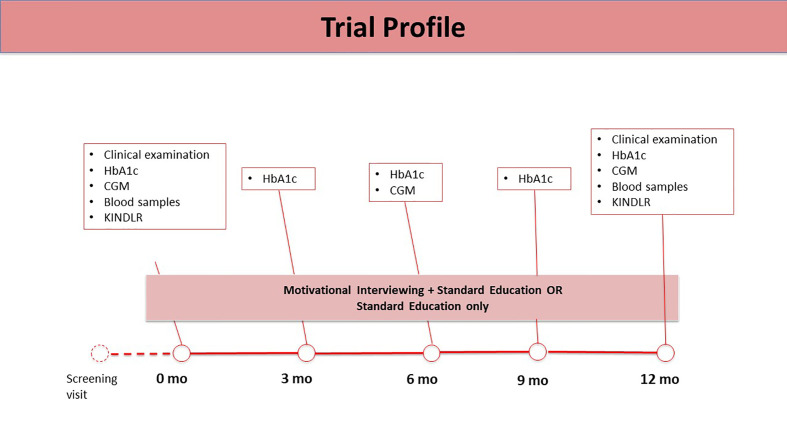
Trial Profile.

The outpatient clinic follow-up visits were scheduled for every three months, over a 12-month span. **Figure 2** presents the study protocol. At each visit, SE included counseling related to the basics of diabetes and carbohydrate counting. All the study physicians had been trained to use SE material that included a slide set describing the principles and goals for treatment of type 1 diabetes, the relationship between glycemic control and micro-vascular complications, and general management principles for hypoglycemia and hyperglycemia. Six of the 12 physicians were randomized to the motivational interview group (MI+SE) and, accordingly, attended a training workshop on provision of MI, run by two experts in the field (M. L. and M. T. T.). Rollnick´s book about MI in health care served as a textbook on the method (13), with special attention to the four core principles of MI: expressing empathy; developing a sense of the discrepancy between *status quo* and desired state; rolling with resistance, as a natural phenomenon; and supporting self-efficacy. In addition, the physicians were provided with written instructions (compiled by M. L.) that included examples of open-ended questions and comments in accordance with MI principles. For the intervention group, the discussion of the educational items at each patient visit incorporated MI principles. The MI focused on improving adherence to glucose monitoring and insulin administration and had the overall aim of improving glycemic control.

### Anthropometrics and Puberty

Subject height, assessed with an electronic stadiometer (Seca GmbH & Co. KG, Hamburg, Germany), was measured to the nearest 0.1 cm. An electronic scale (Seca 770, Seca GmbH) measured patients’ weight to the nearest 0.1 kg. Hip and waist circumference were obtained, *via* a tape measure, to the nearest 0.5 cm. *Z*-scores for child height and BMI were derived from recent Finnish reference material (14), and the IOTF criteria (15) determined the overweight and obesity thresholds. Pubertal Tanner stage was assessed at baseline.

### The Number of Glucose Measurements and Continuous Glucose Monitoring

The number of glucose measurements was counted from the number of fingerpricks (75% of the patients) or number of iCGM scans (25%) during the two weeks prior to study visits. Continuous glucose monitoring (CGM) was performed at baseline, six months, and one year (iPro2 Professional continuous glucose monitor; Medtronic Diabetes, Northridge, CA, USA, or the patient’s own CGM device: Medtronic Enlite in a Veo or 640G insulin pump, by Medtronic Diabetes, or FreeStyle Libre, from Abbott Diabetes Care, Inc.) for six days to measure interstitial glucose levels. Data were available for 11 MI+SE and 18 SE patients at baseline, and for 14 MI+SE and 16 SE patients at 12 months. Complete baseline and 12-month CGM recording data were available for 22 patients (9 MI+SE and 13 SE). To assess glycemic variability, we used mean glucose level; the standard deviation of blood glucose values; the calculated coefficient of variation (SD/mean); and time in range (TIR), defined as blood glucose between 3.9 and 10.0 mmol/l, all from sensor CGM curves (16).

### Blood Work and Laboratory Measurements

Blood work and routine laboratory assessments were performed in conjunction with standard hospital procedures and quality control. On every visit, immediate point of care HbA1c levels were measured from fingertip samples (Afinion™, Abbott, Chicago, USA) (17).

### KINDL-R

Health-related quality of life (HRQL) was assessed during outpatient clinic visits with the German KINDL-R questionnaire, which is available in the Finnish language (18). Versions have been developed for children and adolescents aged 8–11 (Kid-KINDL) and 12–16 (Kiddo-KINDL). This study used the latter, which has 24 items, distributed over six domains: “Physical well-being” (WB), “Psychological well-being”, “Self-esteem”, “Family”, “Friends”, and “School”. In addition, we used modules for chronic disease and diabetes. The KINDL-R instrument employs a Likert scale with five options, and the score range is 0 to 100. A higher score points to better health-related quality of life: the more positive the number calculated, the more positive was the change in the HRQL domain. Results were analyzed with KINDL analysis files (http://kindl.org/english/scoring/).

### Statistics

Data are presented as mean values (and standard deviation) unless otherwise stated. The primary outcome was the change in HbA1c and glycemic variability, evaluated by means of CGM, between baseline and 12 months. Change in HRQL was a secondary outcome measurement. For power calculations, we applied a threshold of 1.0% for a clinically significant mean difference in HbA1c change between the treatment groups, and the calculations used an SD of 1.24, based on our previous experience. For a power of 80% and alpha of 0.05, the study needed at least 50 patients.

Differences in primary outcome measurements (for HbA1c, TIR, and the coefficient of variation) were evaluated *via* univariate ANCOVA with entering treatment as a fixed factor and the baseline as a covariate in the analyses and also through repeated-measures ANOVA with group assignment as the between-subjects factor. On account of skewed distributions, we used log-transformed data for analyses of HbA1c levels. For differences in KINDL outcome measures, we utilized univariate ANCOVA and ANOVA, in the aforementioned manner. We assessed associations between parameters *via* Pearson or Spearman correlations, as appropriate. *P* < 0.05 was considered statistically significant. All analyses were performed with SPSS Statistics, version 25.

## Results

Forty-six patients completed the baseline visit, and 43 of them (93%) completed all study visits. Baseline characteristics were similar between the MI+SE and the SE-only group (see **Table 1**).

**Table 1:** Characteristics of study subjects at baseline, presented as mean values (SD), except for the number of glucose measurements (median).

	MI+SE	SE only
Number	24	23
Male/female sex (*n*)	14/10	12/11
Age (y)	14.6 (0.9)	14.6 (0.8)
Diabetes duration (y)	8.3 (3.8)	7.8 (3.9)
Height (cm)	167.8 (7.2)	167.4 (6.2)
Weight (kg)	65.1 (12)	61.7 (13)
BMI (kg/m2)	23.1 (3.7)	22.0 (4.4)
HbA1c (mmol/mol)	89 (15)	86 (15)
TIR (%)	36 (20)	34 (13)
Continuous sc insulin infusion	15	15
Multiple daily insulin injections	9	8
Two-week mean glucose (mmol/l)	12.1 (2.1)	11.7 (2.7)
No. of glucose measurements in the previous 2 weeks	42	47

### HbA1c, Mean Glucose, Time in Range, and the Coefficient of Variation

**Figure 3:**
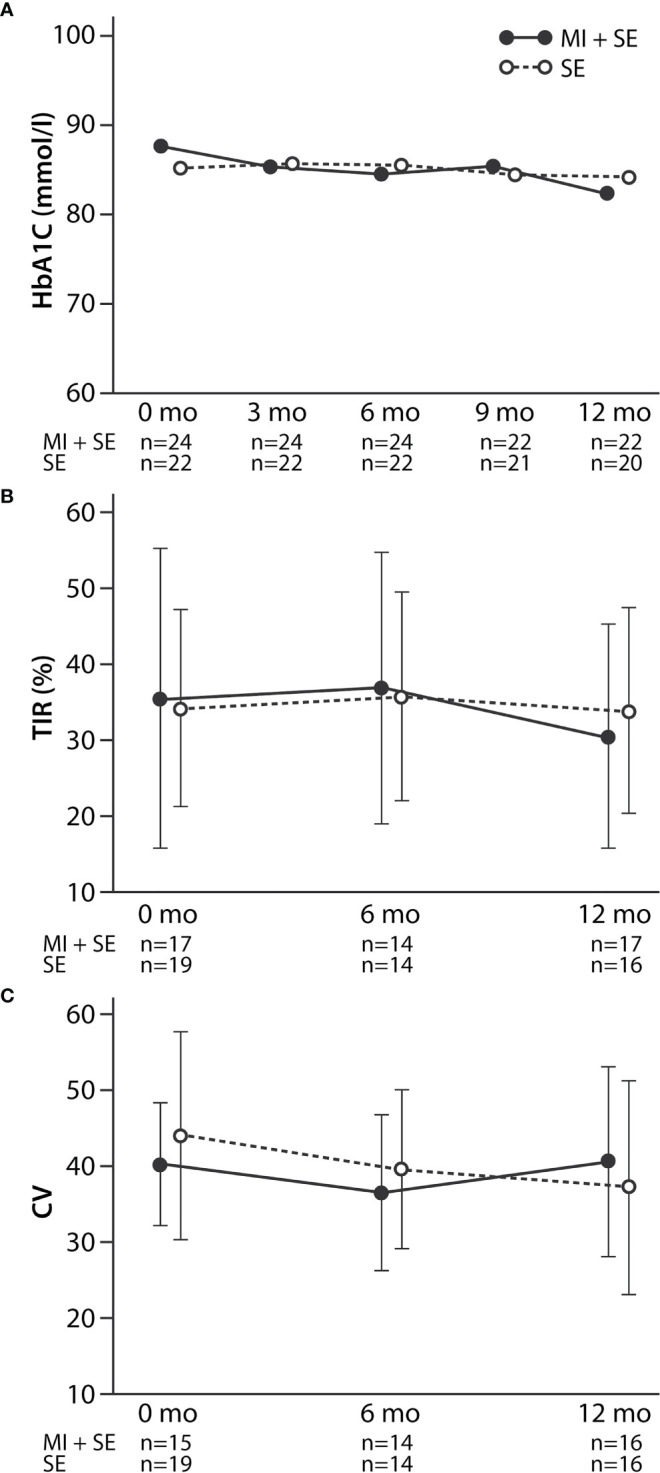
HbA1c **(A)**, time in range (TIR) **(B)**, and coefficient of variation (CV) **(C)** in the MI+SE (motivational interview and standard education) and the SE (only standard education) group during the study, expressed as geometric means (HbA1c) or arithmetic means (TIR and CV). Whiskers indicate SDs. As evaluated by repeated-measures ANOVA, the HbA1c, time-in-range, and coefficient-of-variation levels did not differ between the groups during the study (*P* = 0.15–0.81).

For the MI+SE and SE-only study groups combined, HbA1c did not change significantly over the 12-month study period (-2.4 [95% CI -8 to 3.2] mmol/mol; *p* = 0.39, corresponding to an effect size of 0.16 [95% CI -0.2 to 0.5]). However, baseline HbA1c correlated negatively with 12-month HbA1c change (*r* = -0.61; *p* < 0.001); all patients with a baseline HbA1c value above 90 mmol/mol (*n* = 14) improved their glycemic control during the 12 months of follow-up independently of the study group assignment. The mean baseline-adjusted HbA1c change at 12 months was similar between the MI+SE and SE-only group, at -3.6 (95% CI -9.9 to 2.6) and -1.0 (95% CI -7.6 to 5.5) mmol/mol, respectively (*P* = 0.57), and HbA1c levels during the study did not differ between the groups in repeated-measures analysis (see **Figure 3**). Similarly, the mean 12-month changes in HbA1c within the MI+SE and the SE-only group were not significant (effect size 0.29 [-0.2 to 0.8] and 0.003 [-0.5 to 0.5], respectively). **Table 2** presents numeric data for mean HbA1c at the various points in time.

**Table 2:** HbA1c levels at baseline and three, six, nine, and 12 months (all figures are means +/- SD), showing no significant differences between groups at any point (*P* = 0.43–0.77), although the MI+SE group’s figure seemed to be lower than the baseline at all measurement points.

Time point	MI + SE group	SE-only group	Mean difference between groups for change from baseline
Baseline (*n* = 46)	89 (15)	86 (15)	
3 months (*n* = 46)	87 (17)	87 (13)	2.3 (3.8)
6 months (*n* = 43)	86 (16)	87 (17)	3.5 (5.0)
9 months (*n* = 43)	87 (17)	86 (14)	1.4 (4.8)
12 months (*n* = 42)	84 (15)	86 (15)	4.5 (5.6)

The mean glucose level derived from two-week CGM data did not change over the full study period in either group. The MI+SE group’s mean glucose figure at baseline and at 12 months was 12.1 +/- 2.1 mmol/l and 12.5 +/- 2.5 mmol/l, respectively, and the SE-only group’s corresponding figures were 11.7 +/- 2.7 mmol/land 12.5 +/- 1.8 mmol/l (*P* = NS for within-group changes).

**Figure 4:**
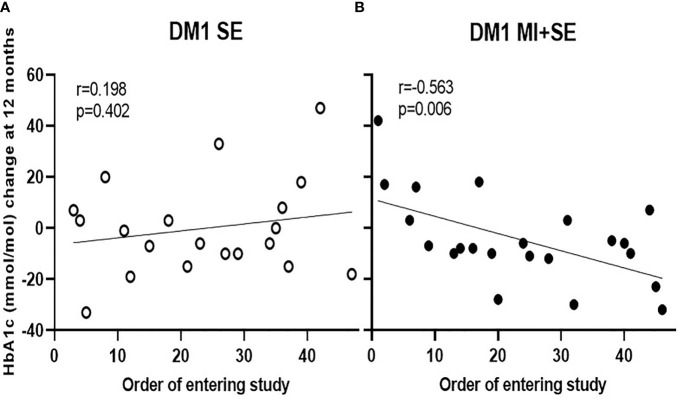
Correlation between the order of joining the study and the 12-month change in HbA1c for the MI+SE **(B)** and the SE-only group **(A)**. Patient 1 was the first patient recruited for the study, and patient 47 was the last.

Further, we found no differences between the groups in TIR or the coefficient of variation as evaluated by comparing mean adjusted 12-month changes (**Table 3**) and repeated-measures analyses (**Figure 3**). Sex had no influence on the mean 12-month change in HbA1c in the MI+SE group (-4.1 mmol/mol for boys and -0.9 mmol/mol for girls; *P* = 0.69) or in the SE group (0.2 and 1.5 mmol/mol, respectively; *P* = 0.89). Neither did pubertal stage or treatment modality (multiple daily injections or continuous subcutaneous insulin infusion) at baseline (*P* = 0.46–0.95). As expected, changes in HbA1c during the study correlated inversely with TIR (*r* = -0.45; *P* = 0.019), independently of group assignment. Time in hypoglycemia displayed no difference between groups (data not shown). However, patient recruitment order did correlate with the 12-month change in HbA1c for MI+SE (*r* = -0.50; *P* = 0.006) and not in the SE-only group (*r* = 0.20; *P* = 0.4) (see **Figure 4**).

**Table 3:** Mean baseline-adjusted 12-month changes in HbA1c, time in range, and coefficient of variation for the MI+SE and the SE group.

	MI+SE	SE	Mean difference	CI 95%	*P-*value
Change in HbA1c (mmol/mol) *n* = 22 for MI+SE and 21 for SE	-3.64	-1.05	-2.59	-6.51 to 11.69	0.568
Change in TIR (%) *n* = 13 for MI+SE and 14 for SE	-0.77	2.63	-3.40	-7.63 to 14.42	0.531
Change in CV *n* = 12 for MI+SE and 14 for SE	-0.50	-6.22	5.72	-15.86 to 4.42	0.255

### The Number of Glucose Measurements

The primary aim behind the intervention (MI+SE) was to improve treatment adherence. For both study groups, we calculated the number of glucose measurements (fingerprick or iCGM scans) within the two-week period preceding each visit. These counts showed no significant changes from baseline in either group at any time point. The number of measurements did not differ between the MI+SE and SE-only group either at baseline (which had a median of 42 and 47 measurements, respectively) or at the 12-month point (with 46 and 45 measurements).

### Health-Related Quality of Life

**Figure 5:**
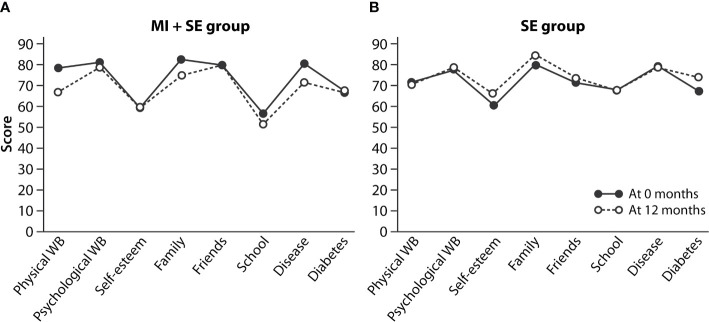
Health-related quality of life, evaluated in Kiddo-KINDL terms, for the MI+SE group **(A)** and SE-only group **(B)** at baseline and 12 months. The values are means.

At baseline, the MI+SE and SE-only group did not show a difference in total KINDL score (118.5 and 121.6, respectively; *P* = NS) or for the KINDL subdomains (see **Figure 5**). Scores in various subdomains were interrelated: the diabetes domain displayed a correlation with scores in the chronic-disease domain (*r* = 0.7; *P* < 0.001), psychological well-being domain (*r* = 0.445; *P* < 0.05), and family-relations domain (*r* = 0.44; *P* < 0.05), and correlation was visible also between the psychological well-being domain and those of self-esteem (*r* = 0.47; *P* < 0.01), family relations (*r* = 0.42; *P* < 0.05), and relationships with friends (*r* = 0.63; *P* < 0.001). Neither the total score nor scores in specific KINDL domains correlated with HbA1c, except for a positive correlation between physical well-being and HbA1c (*r* = 0.41; *P* < 0.05). Accordingly, baseline TIR showed no statistically significant association with any of the KINDL measurements (*P* = 0.08–0.85). Similarly, 12-month changes in HbA1c or TIR were not correlated with KINDL parameters (data not shown). The groups were similar in their mean baseline-adjusted KINDL changes, apart from KINDL School (14.81 to 12.30, with a difference of 0.201–4.803 for the 95% CI), *F* = 5.016 (*df* = 1); *P * = 0.034. No significant changes in any of the scores over the course of the study were visible for either group (see **Figure 5**).

## Discussion

The current RCT´s central premise was that low treatment motivation is a crucial barrier to satisfactory adherence to treatment, and that addressing this through MI would improve glycemic control in adolescents with poorly controlled type 1 diabetes. Contrary to our hypothesis, adding MI to standard care for 12 months of follow-up did not improve HbA1c, TIR, or glycemic variability (CV). This study result is disappointing, in that higher HbA1c levels predict long-term complications (19) and more recent studies imply that low TIR and high glycemic variability predict diabetes-associated mortality (20, 21).

Optimal glycemic control in childhood should be our goal for guaranteeing a low risk for future micro-vascular complications, even in a setting of poor glycemic control later on (22, 23). Reduced treatment adherence is common during adolescence. It may present as acute deterioration, including diabetic ketoacidosis or severe hypoglycemia, and may impact long-term risk of severe complications such as retinopathy, nephropathy, peripheral neuropathy, and cardiovascular disease (24). It is conceivable that an intervention resulting in improved treatment adherence and glycemic control during adolescence could potentially result in sustained beneficial effects and decreased long-term morbidity in type 1 diabetes patients.

There have been few studies of the use of MI in treating diabetes in youth (7, 8, 10, 11). To our knowledge, ours is the only RCT in which the treating physicians employed the method, and there was comprehensive assessment of glycemic control, with TIR and CV in addition to HbA1c measurements. In a recent RCT by Mayer-Davis and colleagues (11), 258 patients were randomized to receive either MI-based counseling or the usual care given by a member of the diabetes team, for 18 months. They found no difference in HbA1c between the groups at 18 months (11). Christie (9) and Wang (10) reported similar results.

Contrasting findings emerged in a study by Channon and colleagues. When 66 teenagers were randomized to receive MI-centered care or the usual care for 12 months, the MI group’s mean HbA1c was significantly lower than the control group’s at 12 months, and also at 24 months, suggesting a sustained benefit of MI (8). The reasons for the contradictory results remain unclear. Thus far, accumulated data has not established an influential role of MI in improving glycemic control in adolescent type 1 diabetes.

Previous studies show no impairment in the general quality of life in patients with type 1 diabetes in comparison to a matched healthy population (25–27). In a study from Germany using the KINDL-R questionnaire, neither this nor general health status was inferior to that of the general population among 11–17-year-old patients with early-onset type 1 diabetes (25). Also, in previous studies on young adults with type 1 onset in childhood who did not exhibit diabetes-related chronic complications, health-related quality of life was similar to that of healthy age- and gender-matched peers (28). However, the combination of mental-health problems and type 1 diabetes is associated with lower quality of life among adolescents compared with the general adolescent population with mental-health problems only (29). Also, there is also evidence suggesting that having both depressive symptoms and diabetes distress is related to suboptimal HbA1c (30). In our study, addressing a population with suboptimal glycemic control, we did not see any significant improvement or worsening of the patients’ quality of life during the study follow-up.

That MI was provided by experienced pediatricians and pediatric endocrinologists instead of other health-care professionals is considered the greatest strength of our study. Furthermore, not just the patients but also the physicians were randomized to the MI+SE or SE-only group, for minimization of differences stemming from such factors as physician characteristics or ways of interacting. The negative association between the order of subjects entering the study and the 12-month change in HbA1c in the MI+SE group suggests that practical MI experience matters. Patients joining the study at the end of the recruitment period, thereby meeting with clinicians who had gained at least some experience in the MI method, seemed to benefit more from the intervention. Therefore, more comprehensive MI training and greater experience in using MI could show a link to improved glycemic control.

The lack of routine psychological evaluation, estimation of the level of parental support in diabetes management, and motivation for glycemic-control improvement were not evaluated in baseline conditions and represent limitations of the current study. Another shortcoming of our study is the small quantity of data on glycemic variability, limiting its statistical power. Further, several different methods were used for continuous glucose monitoring, making us unable to control the putative impact of glucose monitoring methods on the results. Finally, we could not voice record the sessions. Doing so would have allowed us to analyze the combination of MI+SE applied, with particular regard to the physicians’ MI-adherent and MI-nonadherent behavior, and its impact on patients’ motivation (31).

In conclusion, while MI+SE did not show an impact on adolescent type 1 diabetes patients’ glycemic control at 12 months, relative to SE-only patients’, in this randomized controlled multicenter pilot study, experience in the application of MI was associated with improved HbA1c. Also, among patients with inferior glycemic control, both study groups showed an improvement in HbA1c levels, indicating that any intervention is helpful for such patients. The anticipated change in a person’s habits is not a quick phenomenon, and it may require more intensive, extended intervention for adolescents. Therefore, the 12 months of follow-up time we used may have been too little for measurable changes in glycemic parameters to appear. We plan to continue our patients’ follow-up, to study the longer-term effects of the MI intervention on HbA1c. In future, the possible benefits of MI in treating type 1 diabetes patients need to be tested in a larger patient cohort. It would be especially interesting to compare MI with the values work that forms part of acceptance and commitment therapy. Also, using digital tools rather than a physical outpatient setting may serve millennials better in the search for treatment motivation.

## Author Contributions

A-KT, MH, MT, and M-AP designed the study. A-KT, MH, MO, KK, PT, and M-AP were responsible for the study subjects’ enrollment and the collection of data. A-KT, MH, PM, TL, KW, SK, PA, MO, KK, PT, RL, and M-AP met with the patients during the study. A-KT, MH, and M-AP analyzed the data and prepared the first version of the manuscript, with important input from TS. ML provided training in the MI method for the study physicians. All authors contributed to the article and approved the submitted version.

## Funding

The study was supported by grants from the Finnish Foundation for Pediatric Research, the Diabetes Research Foundation, the Sigrid Juselius Foundation, the Medical Society of Finland, and Helsinki University Hospital Pediatric Research Center.

## Conflict of Interest

The authors declare that the research was conducted in the absence of any commercial or financial relationships that could be construed as a potential conflict of interest.
